# Enhancing healthcare outcome with scalable processing and predictive analytics via cloud healthcare API

**DOI:** 10.3389/fdgth.2025.1687131

**Published:** 2026-01-09

**Authors:** Seyede Sana Salehi, Hamid Saadatfar, Solomon Sunday Oyelere, Sadiq Hussain, Javad Hassannataj Joloudari, Mohammad Taheri Ledari, Emrah Arslan, Behnam Barzegar

**Affiliations:** 1 Department of Computer Engineering, Islamshahr Branch, Islamic Azad University, Islamshahr, Iran; 2 Department of Computer Engineering, Faculty of Electrical and Computer Engineering, University of Birjand, Birjand, Iran; 3 Department of Computer Science, Faculty of Environment, Science and Economy, University of Exeter, Exeter, United Kingdom; 4 Department of Computer Science, Electrical and Space Engineering, Luleå University of Technology, Luleå, Sweden; 5 Research Group on Data, Artificial Intelligence and Innovations for Digital Transformation, Johannesburg Business School, University of Johannesburg, Johannesburg, South Africa; 6 Centre for Computer Science and Applications, Dibrugarh University, Dibrugarh, India; 7 Department of Computer Engineering, Technical and Vocational University (TVU), Tehran, Iran; 8 Department of Computer Engineering, Bab.C., Islamic Azad University, Babol, Iran; 9 Department of Computer Engineering, Faculty of Engineering, KTO Karatay University, Konya, Türkiye

**Keywords:** cloud healthcare API, data privacy, machine learning, predictive analytics, scalable processing

## Abstract

This systematic literature review investigates the Google Cloud Healthcare API's role in transforming healthcare delivery through advanced analytics, machine learning, and cloud-based solutions. The study examines current features of cloud-based healthcare platforms in managing heterogeneous healthcare data formats, analyzes the effectiveness of cloud solutions in enhancing clinical outcomes, and compares Google Cloud Healthcare API with alternative platforms. The findings reveal that Google Cloud Healthcare API demonstrates notable advantages through its fully managed, serverless architecture, native support for healthcare standards (e.g., FHIR, HL7v2, DICOM), and seamless integration with advanced AI/ML services. Cloud-based predictive analytics platforms have proven effective in reducing hospital readmissions, addressing physician burnout, and enabling scalable telemedicine solutions. However, significant challenges persist including data privacy concerns, regulatory compliance complexities, infrastructure dependencies, and potential vendor lock-in risks. The research demonstrates that healthcare organizations implementing comprehensive cloud-based solutions achieve measurable improvements in patient outcomes, operational efficiency, and care delivery models. While technical challenges around latency in medical imaging and interoperability remain, the evidence strongly supports cloud adoption for healthcare transformation, provided organizations address security, compliance, and implementation challenges through strategic planning and comprehensive change management approaches.

## 1 Introduction

The modern healthcare industry is undergoing a significant transformation, driven by an exponential surge in the volume, variety, and velocity of data. This phenomenon, often termed “big data,” stems from an increasingly diverse array of sources (1). Electronic Health Records (EHRs) are now considered a cornerstone of clinical practice in healthcare data management. Following widespread digital transformation efforts, EHRs contribute a wealth of structured and unstructured information. This includes detailed patient histories, physician's notes, laboratory results, medication regimens, and diagnosis codes. Simultaneously, different medical imaging modalities such as Computed Tomography (CT), Magnetic Resonance Imaging (MRI), Positron Emission Tomography (PET), and digital pathology are generating vast, high-resolution visual datasets, often measured in terabytes per patient study, which hold immense diagnostic potential but also pose significant storage and processing challenges. Compounding this complexity is the rapid proliferation of wearable devices and Internet of Things (IoT) sensors ranging from continuous glucose monitors and smartwatches to sophisticated remote patient monitoring systems, which stream continuous physiological and behavioral data, offering novel avenues for personalized health insights and real-time interventions (1, 2).

This rich tapestry of information is predominantly formatted in established healthcare data standards. Health Level Seven Version 2 (HL7v2) is a legacy messaging standard that remains deeply embedded in many healthcare information systems, facilitating critical data exchange despite its complexities and limitations. The popular international standard for handling, storing, and transmitting medical imaging information is Digital Imaging and Communications in Medicine (DICOM) that ensures consistency across imaging equipment and software. More recently, Fast Health Interoperability Resources (FHIR) has emerged as a modern, web-centric standard that is also compatible with previous HL7 standards. Leveraging RESTful APIs and FHIR's Resource-based data structures significantly enhance interoperability, particularly for mobile, cloud, and modular applications. In FHIR, resources are the key components used to represent core healthcare data structures format exchange such as patients, practitioners, laboratory results, and code systems. The specification currently defines around 160 resources, with the flexibility to extend and create additional resources as needed^1^. The culmination of these multi-modal data streams, standardized to varying degrees, offers significant opportunities to improve patient care and streamline operational workflows (3). Concurrently, healthcare organizations can streamline operational workflows by optimizing resource allocation, reducing preventable hospital readmissions, improving patient throughput, automating administrative tasks, and enhancing supply chain efficiency. However, the path to realizing these benefits is fraught with substantial challenges. The sheer volume of data strains traditional on-premise storage and computational infrastructure. Its diversity, encompassing structured clinical data, unstructured textual notes, and complex image files, complicates integration and necessitates sophisticated analytical approaches. Particularly, due to the great sensitivity of clinical data that contains Personally Identifiable Information (PII) and Protected Health Information (PHI), stringent security and privacy measures are mandated. These challenges are further intensified by rigorous regulatory frameworks, most notably the Health Insurance Portability and Accountability Act (HIPAA) in the United States, which imposes strict rules regarding the privacy, security, and breach notification of patient data (4, 5). Addressing these issues requires scalable, secure, and innovative technological interventions capable of unlocking the data's potential while upholding the highest standards of confidentiality and regulatory compliance (6). The Healthcare API as a platform-as-a-service (PaaS), bridges traditional healthcare systems with cloud environments by supporting standards like FHIR, HL7v2, and DICOM (7, 8).

By integrating with Google Cloud tools such as Cloud Dataflow for real-time data processing, BigQuery for large-scale analytics, and TensorFlow for machine learning (ML), this API enables healthcare organizations to transform complex datasets into actionable insights (9, 10). This integration is pivotal for advancing data-driven healthcare innovations. A key application of this technology lies in predictive analytics, where ML frameworks like TensorFlow leverage FHIR data to identify patterns and predict patient outcomes (11). By leveraging the Cloud Healthcare API, providers can develop models to anticipate clinical events, personalize treatments, and optimize resource use, shifting from reactive to proactive care (12, 13). Such advancements promise improved patient outcomes and operational efficiency (14). The performance and success of these advances depends on efficient data management and processing capabilities. Tools like Cloud Dataflow and BigQuery facilitate real-time and scalable analyses of both structured and unstructured healthcare data, delivering timely insights that are essential for clinical decision-making and operational improvements (15–17). Nevertheless, their effectiveness must be supported by robust privacy measures to address the ethical and legal dimensions of healthcare data use. Cloud computing solutions, particularly the Cloud Healthcare API, implements encryption and de-identification techniques to protect sensitive patient information, ensuring compliance with regulatory frameworks like HIPAA and the General Data Protection Regulation (GDPR) (18–20). These security features ensure that de-identified data can be ethically utilized for research while preserving its analytical value (13). Maintaining a balance between data accessibility and protection is foundational to sustainable healthcare innovation.

This systematic literature review investigates the Google Cloud Healthcare API's role in transforming healthcare delivery through advanced analytics, machine learning, and cloud-based solutions. The core objectives of this study are to:

Evaluate the API's capabilities in managing and integrating heterogeneous healthcare data formats while supporting critical interoperability standards across diverse clinical environments.
Examine the scalability and performance of cloud-based data processing workflows using integrated platform components.
Analyze the effectiveness of cloud healthcare solutions in enhancing clinical outcomes.
Assess the comparative advantages of Google Cloud Healthcare API over alternative cloud platforms.
Investigate the challenges and success factors associated with implementing cloud-based healthcare solutions.
Synthesize emerging trends and future research directions in cloud-based healthcare analytics.

The rest of the paper is structured as follows: Section 2 outlines the search methodology employed, including the search strategy, inclusion and exclusion criteria, and quality assessment protocols. Section 3 presents a comprehensive literature review examining the existing research on healthcare data management, cloud computing applications in healthcare, and predictive analytics implementations. Section 4 provides an analytical discussion of the benefits and limitations of cloud-based healthcare solutions, comparative analysis with traditional approaches, implications for healthcare delivery, and strategies for addressing implementation challenges identified in the literature. Section 5 examines real-world case studies demonstrating successful implementations of the Cloud Healthcare API, followed by an analysis of key ways cloud-based solutions enhance healthcare delivery, including hospital readmission reduction, physician workflow optimization, and telemedicine expansion. Section 6 explores future research directions and emerging trends in cloud-based healthcare analytics, artificial intelligence applications, and predictive modeling technologies. Finally, Section 7 concludes the paper with a synthesis of key findings, recommendations for healthcare organizations considering cloud adoption, and identification of areas requiring further investigation in the evolving landscape of cloud-based healthcare solutions.

## 2 Research methodology

This systematic literature review was conducted to comprehensively examine the current state of cloud-based healthcare solutions, with particular emphasis on the Google Cloud Healthcare API and its applications in predictive analytics. The review process was structured around five following phases: (1) formulating specific research questions; (2) identification of relevant academic databases and development of a comprehensive search strategy; (3) setting inclusion and exclusion criteria for article selection; (4) implementation of quality assessment protocols to ensure the selection of related, high-quality, peer-reviewed publications; and (5) systematic data extraction to address the research objectives. Given the relatively new and developing state of research specifically focused on Google Cloud Healthcare API, the scope was expanded to include broader cloud computing applications in healthcare, predictive analytics implementations, and healthcare data interoperability standards to provide comprehensive contextual coverage. The systematic review was guided by the research questions listed in Table 1.

**Table 1:** Research questions and goals.

Research Question	Goal
What are the current trends and technical specifications of cloud-based healthcare, particularly the Google Cloud Healthcare API, in healthcare data management and interoperability?	To identify and analyze the technical features, supported data standards (FHIR, HL7v2, DICOM), and integration capabilities of major cloud healthcare platforms, with specific focus on Google Cloud Healthcare API's architecture and functionality.
How are cloud-based healthcare services improving healthcare outcomes, particularly in areas such as hospital readmission reduction, disease prediction, and operational efficiency	To evaluate the evidence for clinical and operational improvements achieved through implementation of cloud-based predictive analytics systems, including quantitative outcomes and performance metrics.
What are the primary challenges and limitations associated with implementing cloud-based healthcare solutions, including data privacy, security, interoperability, and regulatory compliance concerns?	To systematically identify and categorize the barriers to cloud adoption in healthcare settings, including technical, regulatory, and organizational challenges reported in the literature.
How do cloud-based healthcare solutions compare to traditional on-premises systems in terms of scalability, cost-effectiveness, and performance?	To conduct comparative analysis between cloud-based and traditional healthcare IT approaches, examining factors such as infrastructure costs, system performance, and adaptability to changing healthcare needs.
What are the key success factors and best practices for implementing cloud-based healthcare APIs and predictive analytics systems in clinical environments?	To identify evidence-based implementation strategies, organizational readiness factors, and technical requirements that contribute to successful cloud healthcare deployments.
What emerging trends and future research directions are evident in the field of cloud-based healthcare analytics and artificial intelligence applications?	To synthesize current research trajectories and identify gaps in the literature that may inform future research priorities and technological developments in cloud healthcare solutions.

### 2.1 Search strategy

The search strategy was designed to identify relevant literature on cloud-based healthcare solutions, predictive analytics in healthcare, and specifically the Google Cloud Healthcare API. The search was conducted through a multi-database search approach to ensure comprehensive coverage of the available literature. The following steps reveal this process.

#### 2.1.1 Step 1: question formalization

The following questions were defined for article selection: (1) What are the main domains/fields of the searched papers (e.g., cloud services in healthcare, google healthcare cloud api)? (2) Where are these papers published (conferences or journals)? (3) What should be the scope and credibility of these papers? (4) When were the papers published?

#### 2.1.2 Step 2: database selection, temporal, and language restrictions

The search was conducted across multiple electronic digital databases. The selection of these databases was guided by their relevance to healthcare informatics, cloud computing, and predictive analytics research domains. This includes PubMed/MEDLINE for biomedical literature, IEEE Xplore for technical and engineering publications, ACM Digital Library for computer science research, Scopus for multidisciplinary coverage, Web of Science for citation analysis capabilities, Springer, Elsevier and Google Scholar for broader academic content. To limit the search, publications from January 2015 to April 2025. Publications were restricted to English.

#### 2.1.3 Step 3: search terms and keywords

The selected articles from all of the listed databases were on the basis of search string through iterative refinement based on preliminary searches. The core search strategy employed four concept groups: (1) Cloud computing terms including “cloud computing,” “cloud healthcare,” “cloud-based healthcare,” “healthcare cloud,” and “cloud infrastructure”; (2) Healthcare API terms including “healthcare API,” “Google Cloud Healthcare API,” “FHIR,” “HL7,” “DICOM,” and “healthcare interoperability”; (3) Predictive analytics terms including “predictive analytics,” “machine learning,” “artificial intelligence,” “healthcare analytics,” “clinical prediction,” and “healthcare AI”; and (4) Healthcare applications terms including “hospital readmission,” “clinical decision support,” “population health,” “telemedicine,” and “remote monitoring.”.

Boolean operators were utilized to create comprehensive search strings that captured the intersection of relevant concepts. The primary search string was: (cloud computing OR cloud healthcare OR “Google Cloud Healthcare API”) AND (healthcare OR medical OR clinical) AND (predictive analytics OR machine learning OR artificial intelligence) AND (API OR interoperability OR FHIR OR HL7). Additional targeted searches were conducted using specific combinations such as “Google Cloud Healthcare API” AND “predictive analytics,” “cloud healthcare” AND “FHIR,” and “healthcare cloud computing” AND “machine learning”.

Given the relatively recent emergence of cloud healthcare technologies and the limited peer-reviewed literature specifically addressing Google Cloud Healthcare API, the search strategy was expanded to include grey literature sources. This included technical documentation from Google Cloud, industry reports from healthcare technology organizations, conference proceedings from healthcare informatics meetings, and white papers from cloud service providers. Professional databases such as the Healthcare Information and Management Systems Society (HIMSS) repository and the American Medical Informatics Association (AMIA) proceedings were also searched.

#### 2.1.4 Step 4: search execution and documentation

The duplicate articles retrieved from different databases were removed and manually filtered through the Zotero citation management software to organize retrieved articles and eliminate duplicates across multiple databases.

#### 2.1.5 Step 5: criteria for inclusion and exclusion

The inclusion criteria were comprehensive academic and technical literature addressing cloud-based healthcare solutions, healthcare APIs (particularly Google Cloud Healthcare API), and predictive analytics applications in healthcare. Acceptable sources included peer-reviewed journal articles, world-class conference proceedings, reputable technical publications, doctoral and master's theses from accredited institutions, academic books, and technical reports published from 2015 to April 2025. Articles were required to discuss cloud computing implementations in healthcare contexts, healthcare data interoperability standards, machine learning applications in clinical settings, healthcare data management and analytics platforms, or empirical studies demonstrating cloud healthcare outcomes. Given the nature of cloud healthcare technologies, high-quality conference papers, technical reports from major cloud providers, and graduate-level research were included to ensure comprehensive coverage of current developments and practical implementations. The exclusion criteria included blog posts, press releases, marketing materials, informal surveys, popular media articles, and publications without full-text accessibility, publications in non-English languages. Articles outside healthcare and cloud computing scopes without reference to cloud services, clinical data standards, healthcare-specific implementations, or medical applications were excluded. Additionally, outdated sources predating 2015 were excluded. Sources discussing theoretical frameworks without practical evidence, implementation details, or measurable outcomes were also excluded to ensure the review emphasized actionable insights and evidence-based practices.

**Figure 1:**
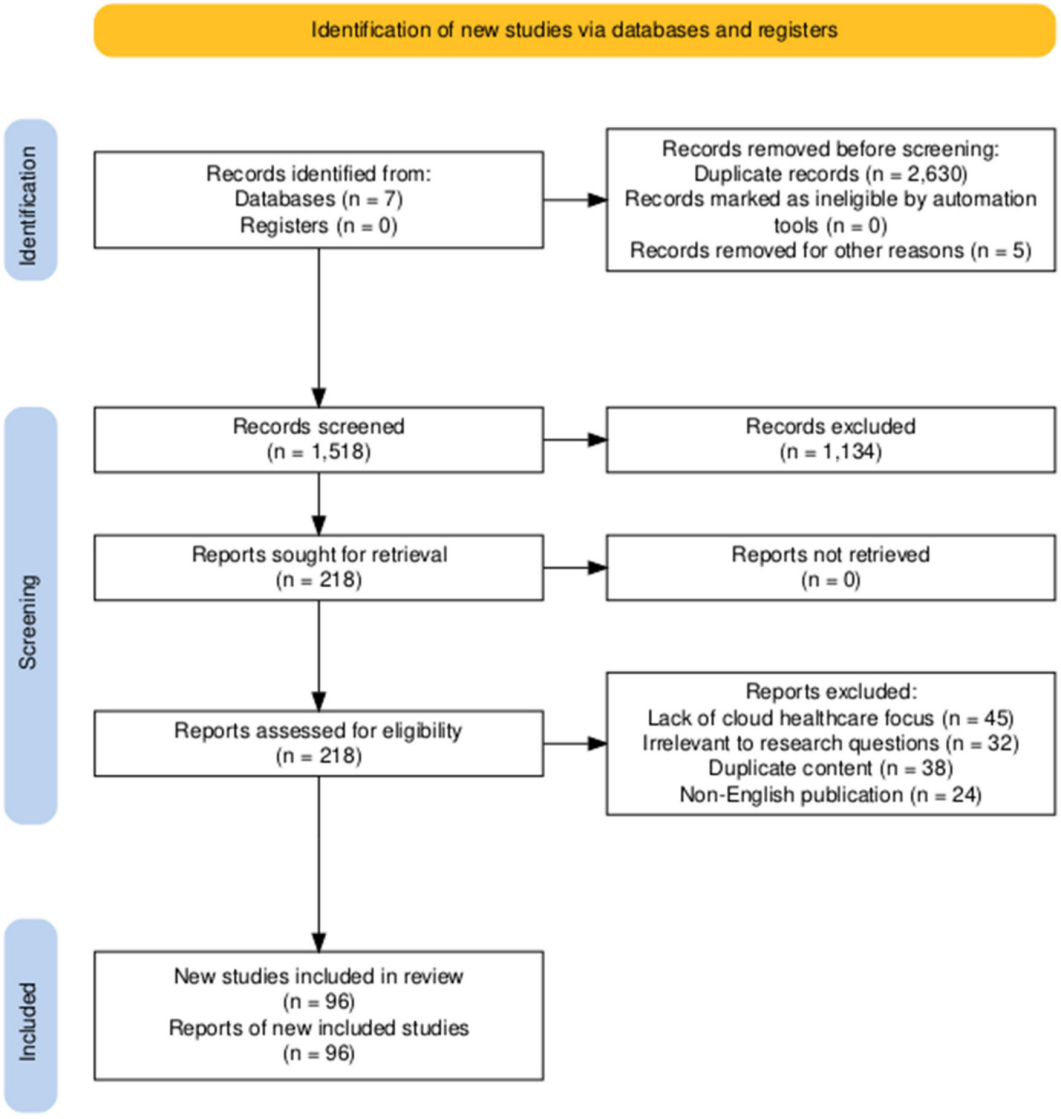
The flow diagram of PRISMA.

After performing the search queries, a total of 4,144 articles from all five major digital databases were retrieved from the initial search. We first applied the duplication criteria, and then set the inclusion and exclusion criteria described in Table 2. Therefore, we first excluded all of the articles found in multiple databases. After removal of duplicates, 1,514 articles remained. In the second phase, we discarded articles published in non-English journals/proceedings, resulting in 1,442 articles for further screening. In the third phase, we excluded articles that were not relevant to the scope. Finally, 380 articles remained for further screening. In the fourth phase, we analyzed the remaining articles on the basis of their title, abstract, and keywords, and the number dropped to 218. In the final phase, after reading and analyzing the full text of the articles, we selected 79 articles from the list to be included in the systematic review. The main steps are outlined in the PRISMA statement in Figure 1.

**Table 2:** Inclusion and exclusion criteria.

Criteria	Inclusion	Exclusion
Publication Type	- Peer-reviewed journal articles - Conference proceedings (IEEE, ACM, HIMSS, AMIA) - Doctoral and Master's theses - Academic books and book chapters - Technical reports from reputable organizations - White papers from major cloud providers	- Blog posts and opinion pieces - Press releases and marketing materials - Popular media articles - Editorial notes and letters - Informal literature surveys - Social media content
Language	- English language publications only	- Non-English publications
Time Period	- Published between 2015- Apr. 2025	- Publications before 2015 - Publications after 2025 up to April 2025
Subject Matter	- Cloud-based healthcare solutions - Healthcare APIs (Google Cloud Healthcare API, AWS, Azure) - Healthcare data interoperability (FHIR, HL7v2, DICOM) - Predictive analytics in healthcare - Machine learning in clinical settings - Healthcare data management platforms - Cloud computing security in healthcare	- General cloud computing without healthcare focus - Healthcare topics without cloud/API discussion - Pure theoretical frameworks without implementation - Topics outside healthcare domain
Accessibility	- Full-text articles available through institutional access - Open access publications - Accessible through academic databases	- Abstract-only publications - Pay-walled content without institutional access - Corrupted or incomplete files
Quality	- Peer-reviewed publications - Theses from accredited institutions - Publications with clear methodology - Evidence-based studies with measurable outcomes - Technical implementations with performance data	- Non-peer-reviewed sources (except technical reports) - Publications lacking methodological rigor - Sources without empirical evidence or case studies - Duplicate publications or republished content
Relevance	- Direct relevance to research questions - Practical implementation examples - Comparative studies of cloud platforms - Performance evaluations and case studies - Security and privacy considerations in cloud healthcare	- Tangential relevance to research questions - Purely historical perspectives without current applicability - Studies focusing on outdated technologies - Generic IT studies without healthcare specificity

The distribution of the articles includes approximately 65% journal articles, 25% conference proceedings, 7% technical reports, and 3% books/chapters. The conferences represented are the main international conferences on health care or health care informatics, whereas the journals represent the world-class reputable journals in the field of computer science and health care. Global representation revealed strong contributions from the United States, which may become a factor that pushes the adoption of the standard in the country and in the rest of the world. In Europe there's a strong focus on GDPR compliance and privacy-preserving healthcare technologies. There is also emerging research on large-scale healthcare digitization in Asia-Pacific.

## 3 Literature review

The rapid digital transformation of the healthcare sector fueled by the merging of cloud computing, big data analytics, and artificial intelligence, offers unprecedented opportunities for improving patient care, operational efficiency, and medical research. In recent years, the adoption of cloud-based platforms has expanded significantly, enabling seamless interoperability and real-time data access across diverse clinical environments. Researchers have explored a wide range of applications, from predictive analytics for reducing hospital readmissions to advanced machine learning models for personalized treatment recommendations, highlighting both the technical potential and operational challenges of these technologies. However, concerns surrounding data privacy, regulatory compliance, system integration, and equitable adoption remain key to ongoing discourse.

### 3.1 Healthcare data management

Healthcare Data Management System (HDMS) has undergone significant change, transitioning from paper-based records to computerized systems, and further advancing through web technologies, cloud computing, IoT integration, big data analytics, and even blockchain solutions to increase security. Today, healthcare data management is fundamentally digital, spanning a wide array of connected medical devices and EHRs that store information either on-premises or in the cloud allowing centralized and secure access to authorized users and healthcare organizations. This digital landscape generates vast amounts of both structured and unstructured data, each presenting distinct management and analytical challenges. Structured data including patient demographics, laboratory results, and billing codes is highly organized and readily accessible through conventional database systems and SQL queries. In contrast, unstructured data such as clinical notes, medical images, and patient-generated content lacks a standardized format, making its processing and analysis more complex. The rapid use of EHRs has significantly increased the availability and diversity of healthcare data, particularly unstructured data. This has driven the adoption of advanced analytics techniques such as natural language processing (NLP) and ML to extract meaningful insights. As healthcare data continues to grow in volume and complexity, the need for scalable, secure, and interoperable data management solutions has become more noticeable (21, 22).

### 3.2 Interoperability standards in healthcare

A critical enabler of healthcare interoperability is the adoption of standardized data formats, particularly FHIR, HL7v2, and DICOM. By enabling secure and efficient information flow, these standards support healthcare providers in delivering timely, data-driven care while upholding strict data privacy and security requirements. FHIR and HL7v2 provide unified frameworks for the seamless exchange, integration, and sharing of electronic health information, supporting effective communication across diverse healthcare systems and applications. This enhanced interoperability is essential for improving care coordination, informed clinical decision-making, and overall healthcare efficiency, ultimately leading to better patient outcomes. Meanwhile, Picture Archiving and Communication Systems (PACS), built on DICOM ensures the standardized and efficient storage, transmission, and sharing of medical images, facilitating interoperability across imaging devices and systems. However, the adoption of these standards have faced significant security challenges, highlighted by incidents of unprotected medical imaging servers and the need for robust safeguards such as encryption, strong authentication, network segmentation, and regular security audits though adoption of such safeguards remains inconsistent across healthcare organizations (23–26).

### 3.3 Cloud computing in healthcare

Cloud computing has emerged as a cornerstone of modern healthcare data management, offering enhanced security, scalability, cost efficiency, and enabling better healthcare delivery, especially when combined with wearable technology and multi-sensor data fusion for real-time monitoring using smartphones and wearables. As highlighted by Awotunde et al., cloud-based solutions allow healthcare organizations to reduce capital expenditures associated with maintaining traditional on-premises IT infrastructure, shifting to more flexible, pay-as-you-go models that scale with demand. This shift not only supports cost savings to up to 30% compared to legacy systems, but also enhances operational agility, allowing providers to rapidly scale resources during peak periods or public health crises without major upfront investments. Furthermore, cloud-based systems facilitate the effective management of EHRs through coordinated information banks, shared EHR platforms, and secure authorization servers. Open-source frameworks like Hadoop are increasingly used to handle the growing volume and diversity of healthcare data, encompassing structured, unstructured, and semi-structured formats (27). Vivekananda et al. highlighted the rapid growth of healthcare, driven by expanded coverage, service supply, and investments from both public and private sectors. They identified the challenges posed by aging populations and the need for innovative, cost-effective solutions (28). Reviewed papers about adopting cloud-based technologies have demonstrated improved efficiency, accuracy, and accessibility across the healthcare sector. In this regard, Varun Shah and Sreedhar Reddy Konda (29), cloud computing streamlines operations, enhances access to patient data, and fosters collaboration among care teams, while also offering scalability and flexibility for adapting to changing demands. One study, Sadoughi et al. (30), systematically reviewed the factors influencing cloud adoption in healthcare, emphasizing the importance of identifying technological, organizational, environmental, and individual factors to ensure successful implementation and maximize benefits such as improved data storage, sharing, and coordination among providers. Another study, Osama et al. (31), examined the integration of cloud computing with existing healthcare systems and found significant improvements in data management efficiency, highlighting how cloud platforms simplify data processes and enable easier, more accurate access and sharing of patient information. Furthermore, cloud-based solutions support advanced analytics and big data applications, empowering healthcare organizations to derive actionable insights, predict disease trends, and personalize care.

However, all three studies underscore persistent challenges: data security, privacy, regulatory compliance, and interoperability remain critical barriers to widespread adoption. Addressing these concerns requires robust risk management, adherence to regulatory frameworks such as HIPAA, and the implementation of security best practices including encryption, access controls, and regular audits. Emerging health policies are increasingly focused on achieving ubiquitous, secure access to health data through cloud migration, which promises greater productivity and efficiency while reducing barriers to innovation. Cloud computing's core features such as on-demand self-service, broad network access, resource pooling, rapid elasticity, and measured service give healthcare organizations the scalability and flexibility to manage fluctuating workloads and support advanced analytics, AI, and telemedicine applications. These benefits extend to both large hospital systems and smaller clinics, democratizing access to sophisticated IT capabilities that were previously out of reach for many providers (27, 32).

### 3.4 Security and privacy considerations

Healthcare databases contain highly confidential information that must be protected through comprehensive safeguards. Ensuring data privacy and security is of utmost importance in this sector, especially when leveraging cloud-based tools such as the Cloud Healthcare API for predictive analytics. Healthcare data faces a variety of threats throughout its lifecycle, with network and storage attacks being common across all types of data. Unique to healthcare, reconstruction attacks and history manipulation are particularly prominent (33). To handle privacy issues, Elayan et al. introduced Deep Federated Learning, a privacy-preserving framework that secures patient data while optimizing performance and reducing operational costs through IoT-enabled devices (34). Additionally, hybrid deep learning techniques have shown promise in improving disease diagnosis accuracy and efficiency, underscoring the transformative potential of ML in healthcare (35). However, integrating ML and deep learning (DL) into real-world healthcare applications presents significant challenges. Ensuring safety requires models to perform reliably not just in controlled settings but also with rare and subtle health conditions. Privacy remains critical, necessitating robust anonymization and secure data handling.

Ethical considerations demand responsible data use to prevent societal harm, while causal reasoning is essential for fair and effective treatment decisions. Regulatory and policy frameworks must keep pace with technological advances to ensure compliance and safe AI deployment. Additional barriers include limited availability and quality of standardized data, distribution shifts, and the complexity of updating hospital IT infrastructure, all of which hinder effective model training and integration (36–43).

In the realm of ML systems, security threats can be grouped into three main categories (44). Firstly, influence attacks that seek to manipulate ML models either by controlling training data (causative) or exploiting model vulnerabilities (exploratory). Secondly, security violations that target the availability and integrity of services, including integrity attacks (false negatives), availability attacks (false positives), and privacy violations. Thirdly, threats that range from targeted attacks on specific inputs to indiscriminate attacks causing widespread failures. These challenges highlight the need for robust, secure, and ethical integration of ML/DL in healthcare to fully realize their benefits (43).

The Cloud Healthcare API enables organizations to implement advanced security measures, including encryption and strong authentication protocols, to effectively safeguard sensitive data. Additionally, the API offers a centralized platform for managing privacy settings and consent preferences, adaptable to various regulatory frameworks and consent standards (17). A critical element in strengthening data privacy in healthcare involves adhering to regulations such as HIPAA, 21 CFR Part 11, PIPEDA, and other global compliance standards. The Cloud Healthcare API is specifically designed to meet these regulations and undergoes rigorous assessments for security, privacy, and compliance. This ensures that healthcare institutions can confidently adopt the API while maintaining the integrity, confidentiality, and security of their data (18). Moreover, the Cloud Healthcare API provides de-identification capabilities that mask or remove protected health information. De-identified data can be safely used for machine learning applications, advanced analytics, training, and sharing with authorized entities. By segregating electronic PHI from de-identified data through API features, organizations can appropriately safeguard sensitive information while still enabling valuable insights and analytics (45). In an environment where data breaches are increasingly common and regulatory scrutiny is intensifying, leveraging cloud-based solutions like the Cloud Healthcare API is essential. With its robust security measures, encryption protocols, and compliance capabilities, the API helps healthcare organizations protect sensitive information while supporting secure, compliant extraction of meaningful insights to predict and improve health outcomes (46).

### 3.5 Predictive analytics in healthcare

The rise of predictive analytics in the healthcare industry has garnered significant attention due to its potential to transform healthcare delivery dramatically and improve both health and financial outcomes. As digital medical records continue to grow exponentially, healthcare institutions are faced with the challenge of managing vast amounts of data in diverse forms at unprecedented rates (47). Clinical data serve multiple purposes in healthcare analytics, including providing a foundation for collecting EHR data, performing data analysis, visualizing insights, and offering clinical decision support. The primary objective of healthcare analytics is to utilize this data to make informed decisions, identify patterns and trends that can enhance patient outcomes, streamline operational processes, and reduce costs (48). Predictive analytics play a pivotal role in forecasting various clinical variables using healthcare data. By examining patterns within these vast datasets, healthcare providers can pinpoint areas for improvement, make more accurate predictions about health outcomes, and tailor care plans to individual patients. Predictive analytics applications include managing population health, supporting clinical decision-making systems, monitoring diseases, and enhancing quality improvement efforts (49). The field of healthcare analytics continues to evolve with the continuous emergence of new technologies. Progress in hardware and software technologies has led to a focus on using machine learning models to extract insights from vast amounts of available healthcare data. Harnessing predictive analytics enables proactive steps toward preventive care and promoting population health management (50, 51). In essence, predictive analytics is a vital component of contemporary healthcare systems, aimed at improving patient outcomes while optimizing operational efficiency. The convergence of big data, advanced analytics, and cloud computing platforms creates unprecedented opportunities for healthcare transformation. Utilizing cutting-edge tools such as the Cloud Healthcare API combined with machine learning algorithms on cloud platforms has great potential for enhancing personalized treatment plans, achieving precise diagnoses through predictive models, and developing more effective preventive care strategies. As healthcare continues to generate increasingly complex and voluminous data, the role of predictive analytics will become even more critical in unlocking the full potential of healthcare big data to improve patient care and population health outcomes (1, 52).

## 4 Overview of the cloud healthcare API

The healthcare sector, though a relatively recent adopter compared to industries like banking or e-commerce, is rapidly embracing cloud computing to meet the demands of big data. Major cloud service providers such as Amazon Web Services, Microsoft Azure, and Google Cloud offer platforms that support the storage, management, and analysis of massive healthcare datasets. These solutions enable enhanced diagnosis, predictive analytics, personalized medical care, and operational efficiency, while also reducing costs and improving data transfer speeds. Table 3 compares major cloud healthcare services. Among these, Google Cloud Healthcare API offers wide variety of interoperability features and a robust security framework for its robust interoperability features, and comprehensive security framework. The API serves as a managed service, serverless solution that bridges traditional healthcare systems with Google Cloud's advanced analytics and machine learning capabilities. Google Cloud Healthcare API natively supports key healthcare data standards such as HL7, FHIR's different versions (e.g., DSTU2, STU3, R4), HL7v2, and DICOM, enabling seamless integration and exchange of clinical and imaging data across diverse healthcare systems. The API streamlines data storage and access through cloud-based repositories, allowing users to store and retrieve data in its native FHIR format while providing flexibility to convert other formats, such as CSV or HL7v2, into FHIR using Cloud Data Fusion. Dedicated API endpoints facilitate interactions with DICOM data for medical imaging, while HL7v2 message ingestion is supported through Minimal Lower Layer Protocol (MLLP) adapters (16, 17).

**Table 3:** Comparison of Major cloud healthcare services.

Feature	Google Cloud Healthcare API	AWS Healthcare Services	Azure Healthcare Services
Supported Standards	FHIR, HL7v2, DICOM	FHIR, HL7v2, DICOM	FHIR, HL7v2, DICOM
Service Model	Fully Managed, Serverless	Managed & Deployable Services	Managed PaaS
Scalability	Automatic Elastic Scaling	High Elasticity with Customer Scaling	Automatic and Manual Scaling Options
AI/ML Integration	Integrated with Vertex AI, AutoML	Integrated with SageMaker, Comprehend Medical	Integrated with Azure ML, Cognitive Services
Security Features	Encryption at Rest & Transit, IAM, DLP, De-identification	Encryption, IAM, Artifact, HIPAA Eligible	Encryption, Role-Based Access, Compliance Center
Pricing Model	Pay-As-You-Go	Pay-As-You-Go	Pay-As-You-Go
Interoperability	Strong, Supports Multiple Healthcare Standards	Good, but customer manages some aspects	Strong, Microsoft Ecosystem
Deployment Complexity	Low, API-based	Medium, requires setup and management	Medium, better with Microsoft infrastructure
Data Processing	Real-time streaming & batch processing	Real-time with Kinesis, batch processing	Stream Analytics, batch processing
Healthcare-Specific Features	Native FHIR R4, STU3, DSTU2 support	HealthLake for FHIR transformation	Azure API for FHIR
Industry Compliance	HIPAA, GDPR, HITRUST compliant	HIPAA, HITRUST, GDPR compliant	HIPAA, HITRUST, GDPR compliant
Ease of Use	Developer Friendly REST & RPC AP	Complexity depends on configuration	Familiar for Microsoft Users
Data Import/Export	Bulk FHIR & DICOM import/export	Bulk operations available	Bulk operations with additional setup
Network Performance	Google's private global network	AWS Global Accelerator	Azure Front Door

Google Cloud's ecosystem provides powerful analytics tools, including BigQuery for large-scale data analysis and Dataflow for real-time data processing, alongside comprehensive ML and AI services such Vertex AI to build and manage ML models. AutoML as a service offered within Vertex AI, lets developers automate the process of building and deploying ML models. These capabilities allow healthcare organizations to process petabyte-scale datasets, apply advanced predictive models, and extract actionable insights from both structured and unstructured data, supporting applications from disease prediction and drug discovery to operational optimization and population health management.

Google Cloud Healthcare API is designed with healthcare's stringent privacy and security requirements in mind. Features include encryption at rest and in transit, granular identity and access management (IAM), Data Loss Prevention (DLP) and compliance with critical regulations such as with HIPAA and GDPR. The platform incorporates automated tools for data loss prevention and de-identification, enabling secure data sharing for research and analytics while maintaining patient privacy. Private Google Cloud Buckets and role-based access control (RBAC) ensure that only authorized personnel can access sensitive healthcare information (53–55).

Leveraging Google's global infrastructure, Google Cloud Healthcare API offers high availability, rapid elasticity, and cost-effective storage. This allows organizations to efficiently manage their workloads, support telemedicine and remote monitoring, and scale research initiatives without service interruption. The platform's developer-friendly REST and Remote Procedure Call (RPC) APIs reduce development complexity and time-to-market compared to the more complex configurations often required by AWS services. The API's RESTful and RPC reduce development complexity, learning curve, and time-to-market. Integration of these features with Google's development tools, such as ML capabilities, enable healthcare organizations to automate data ingestion, transformation, and analysis. The platform streamlines critical healthcare workflows, including appointment scheduling, medical image analysis, and remote patient monitoring, making it significantly easier to deploy and scale new digital health services. In the comparative study by Manhas and Hariharan (55), Google Cloud Platform demonstrated suitability for healthcarefor healthcare and life sciences ventures due to its comprehensive healthcare-specific offerings, advanced analytics capabilities, and streamlined development environment.

Comparative studies indicate that Google Cloud Healthcare API's focus on healthcare-specific standards, advanced analytics, and robust security often make it the preferred choice for organizations to gather knowledge from the available information. Its ability to support real-time analytics, seamless interoperability, and secure data sharing empowers providers to drive innovation, improve care coordination, and enhance patient outcomes. However, realizing these benefits requires a strategic approach that addresses data security, regulatory compliance, and ongoing operational challenges inherent in healthcare digital transformation (1, 53–56).

### 4.1 Cloud platform components

The Google Cloud Healthcare API operates within a comprehensive ecosystem of integrated cloud services that collectively enable healthcare data processing, analytics, and machine learning capabilities. This section examines the core platform components for healthcare data transformation and predictive analytics. Cloud Dataflow serves as the backbone for both real-time and batch data processing, utilizing Apache Beam to create unified pipelines that can handle diverse healthcare data streams from EHRs to IoT medical devices. BigQuery functions as the scalable data warehouse solution, providing serverless analytics capabilities. The Cloud Machine Learning Engine offers the computational infrastructure necessary for training and deploying sophisticated predictive models using frameworks such as TensorFlow. Together, these components create a seamless data pipeline that ingests raw healthcare data, transforms it into analytical insights, and powers decision-making systems. Understanding the capabilities and integration points of these platform components is essential for healthcare organizations seeking to leverage cloud-based solutions for improved patient outcomes, operational efficiency, and predictive healthcare delivery.

#### 4.1.1 Cloud dataflow for efficient data processing

Cloud Dataflow is an essential service for managing healthcare data efficiently in cloud environments, specifically designed to manage large-scale healthcare data efficiently by enabling both batch and real-time data processing. Built on Apache Beam, Cloud Dataflow enables healthcare organizations to design unified pipelines that can process diverse data streams ranging from structured EHR entries to unstructured imaging data and continuous sensor streams from wearable and IoT devices while supporting timely clinical and operational decision-making (14) A key advantage of Cloud Dataflow is its seamless integration with other Google Cloud services, allowing it to operate as a central orchestrator in healthcare data workflows. For instance, Google Cloud Pub/Sub is a scalable messaging service that acts as a real-time event broker, enabling the ingestion of data from various sources such as medical devices, EHR systems, and IoT sensors. Pub/Sub collects and delivers streaming data to Dataflow pipelines, allowing for immediate processing and supporting event-driven analytics. This architecture is particularly valuable in healthcare scenarios requiring rapid response, such as patient monitoring and alerting. Dataflow pipelines can then write directly to BigQuery, Google's serverless data warehouse, enabling dynamic dataset creation and manipulation for downstream analytics. This integration supports large-scale data analysis and reporting, making it easier for healthcare organizations to extract insights from heterogenuous data. Apache Beam's unified programming model allows organizations to execute both batch and streaming analytics pipelines, supporting a wide range of healthcare data scenarios, from retrospective studies to real-time clinical decision-making (15). Beyond core data processing, Cloud Dataflow streamlines the process of reading from and writing to BigQuery, facilitating the efficient storage, transformation, and analysis of large volumes of healthcare data. For advanced processing needs, Dataproc clusters can be configured with the BigQuery Connector, enabling additional analytics and machine learning tasks on ingested medical data (16).

Additionally, Cloud Dataflow also supports the development of interactive tools such as Datalab, which provide healthcare professionals with capabilities for data exploration, visualization, and machine learning. These tools enable healthcare professionals to visualize BigQuery data, collaborate with colleagues, and accelerate innovation in healthcare analytics (57). Cloud Dataflow plays a pivotal role in streamlining data processing workflows within healthcare organizations. By leveraging its integration with services like Pub/Sub, BigQuery, and Dataproc, healthcare providers can efficiently manage, analyze, and derive actionable insights from their data. This ultimately leads to improved patient outcomes and enhanced operational efficiency, supporting the ongoing digital transformation in healthcare.

#### 4.1.2 Bigquery for scalable analytics

Leveraging Google Cloud's provides scalable analytics capabilities for healthcare organizations for organizations aiming to extract actionable insights and improve patient outcomes. BigQuery operates as a fully managed, serverless data warehouse that enables users to execute rapid, SQL-like queries on extensive datasets. This capability is particularly valuable in healthcare, where providers must efficiently manage and analyze massive volumes of data, including formats such as FHIR, HL7, and DICOM. A core advantage of BigQuery lies in its ability to handle huge datasets while maintaining high performance. This is essential in healthcare, where the volume and diversity of patient data can be challenging to handle. BigQuery enables organizations to run complex analytics and machine learning algorithms on their data with near real-time responsiveness, supporting timely decision-making and predictive modeling. Its automatic scaling ensures that computational resources are dynamically allocated to meet the demands of each query, allowing users to process terabytes or even petabytes of data without the burden of managing underlying infrastructure or clusters (58) Moreover, beyond its scalability and performance, BigQuery integrates seamlessly with other cloud tools, such as Cloud Dataflow and AI platforms, to facilitate data transformation and visualization. Healthcare researchers and professionals can create interactive dashboards, execute batch analytics pipelines, and perform advanced data transformations, all within a unified ecosystem. This integration supports comprehensive data analysis workflows, from ingestion and processing to visualization and reporting (59, 60).

**Figure 2:**
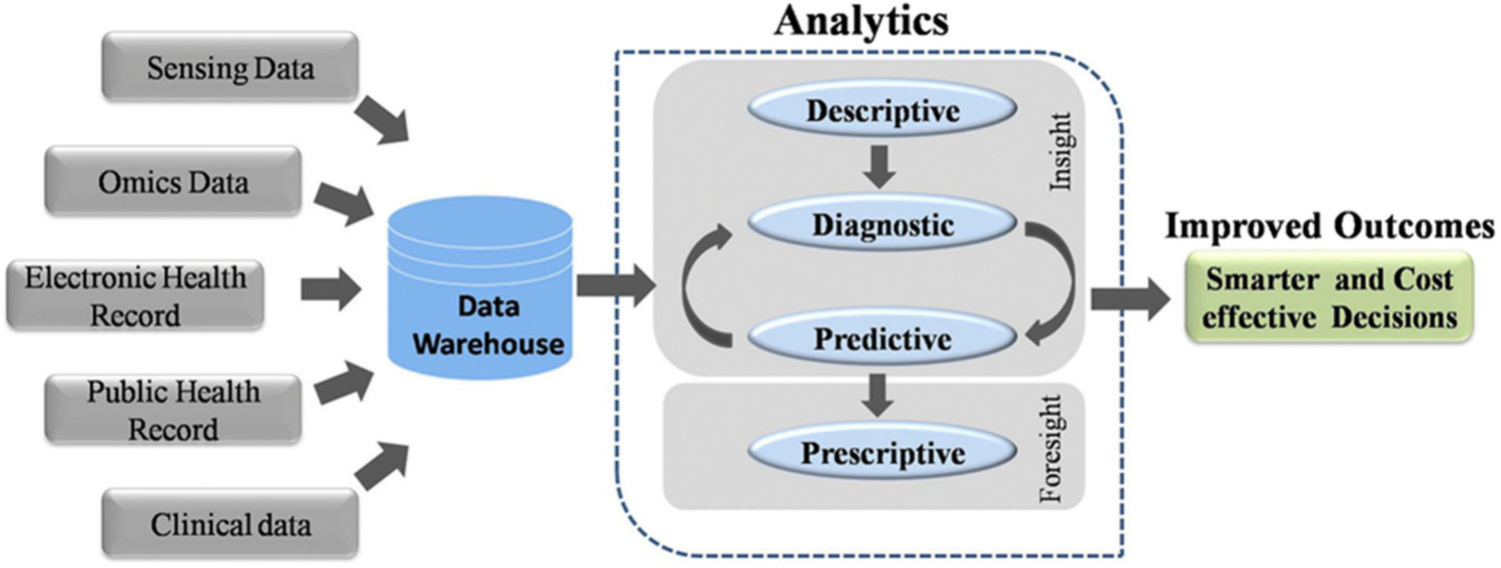
Workflow of Big data analytics in healthcare.

Figure 2 illustrates the workflow of big data analytics in healthcare. Data warehouses collect and store massive amounts of information from various sources, which are then processed through analytic pipelines to enable smarter and more cost-effective healthcare decisions (1).

In conclusion, adopting BigQuery for scalable analytics opens up significant opportunities for healthcare organizations seeking to leverage their data for predictive analytics and operational insights. By incorporating BigQuery into their data strategy, healthcare providers can enhance patient care, optimize resource allocation, and drive innovation across the industry (61).

#### 4.1.3 Leveraging a cloud machine learning engine for advanced machine learning

Utilizing the Cloud Machine Learning Engine for advanced machine learning applications in the healthcare industry introduces a novel approach to predictive analytics, data-driven, and personalized patient care. The integration of artificial intelligence (AI) and ML within cloud environments enables healthcare organizations to analyze massive, heterogeneous datasets ranging from structured EHRs to unstructured clinical notes and imaging at unprecedented scale and speed. In this ecosystem, the Cloud Healthcare API provides a secure, compliant, and interoperable platform that bridges data processing, analytics, and machine learning workflows. Through native support for healthcare data standards such as FHIR, HL7v2, and DICOM, the API ensures seamless data integration from diverse sources, allowing for comprehensive analytics and model development. This interoperability is crucial for training robust ML models that require access to high-quality, multi-modal health data (62).

Importantly, the platform's security architecture addresses the stringent privacy and regulatory requirements of the healthcare sector. The platform's security features such as encryption at rest and in transit, Role-Based Access Control (RBAC), and automated de-identification of sensitive information ensure that patient data can be safely leveraged for advanced analytics and AI applications. This compliance with frameworks like HIPAA and GDPR is essential for fostering trust and enabling broader adoption of cloud-based ML solutions in healthcare environments. Cloud-based machine learning engines, such as those provided by Google Cloud, offer scalable infrastructure that supports the rapid training and deployment of complex models, including deep learning architectures. This scalability allows healthcare institutions to experiment with and operationalize predictive models for a range of applications, from early disease detection to resource optimization and personalized treatment planning. The seamless integration with other cloud services such as BigQuery for data warehousing and Dataflow for real-time data processing further enhances the ability to build end-to-end solutions that are both efficient and adaptable to the clinical needs. Ultimately, leveraging the Cloud Machine Learning Engine in conjunction with the Cloud Healthcare API empowers healthcare organizations to move beyond traditional analytics toward intelligent systems. This approach not only accelerates the development of innovative applications but also democratizes access to advanced AI tools, enabling both large health systems and smaller providers to benefit from predictive analytics, tailored interventions, and improved patient outcomes on a scalable and secure foundation (10).

## 5 Discussion

The implementation of cloud-based healthcare solutions, particularly through the Google Cloud Healthcare API, represents a significant development in healthcare data management and predictive analytics. Cloud-based healthcare solutions deliver substantial benefits across multiple dimensions of healthcare delivery. Enhanced data accessibility and interoperability enable real-time data sharing and collaborative decision-making that breaks down traditional healthcare silos. The scalable infrastructure empowers organizations to efficiently support big data analytics and advanced machine learning applications. Healthcare generates massive heterogeneous data requiring advanced cloud architectures to handle exponential growth of diverse data types (63). Recent advances in interpretable machine learning, including explainable AI techniques for prediction tasks, demonstrate the growing importance of model transparency and interpretability across diverse domains. These methodological developments align with the healthcare sector's need for clinically interpretable predictive models that can support evidence-based decision-making while maintaining stakeholder trust and regulatory compliance.

However, significant limitations persist. Data privacy and regulatory compliance remain critical concerns given the sensitive nature of healthcare information and stringent HIPAA and GDPR requirements. Technical challenges around system integration, data heterogeneity, and model interpretability can inhibit seamless adoption and clinical deployment trust. The digital divide poses important limitations, as smaller healthcare organizations may lack technical expertise or financial resources to fully leverage advanced cloud solutions, potentially exacerbating healthcare delivery disparities.

Traditional healthcare IT infrastructure relying on on-premises systems differs significantly from cloud solutions. Legacy systems suffer from fragmented data silos, limited interoperability, and significant barriers to implementing advanced analytics. Demonstrated that cloud computing environments effectively address critical limitations including lack of scalability, high administrative costs, and inefficient resource utilization (64). Cloud platforms enable seamless collaboration while providing faster deployment, integrated AI capabilities, and robust disaster recovery. However, traditional approaches retain value for highly customized applications or environments with stringent data locality requirements. Hybrid models may offer optimal solutions for organizations with complex regulatory requirements.

Cloud-based solutions fundamentally alter healthcare delivery landscapes. Telemedicine and remote monitoring substantially increase care access, particularly benefiting underserved populations. Data-driven care models enable proactive, personalized interventions enhancing disease prevention and chronic disease management. Cloud platforms streamline clinician workflows through automated documentation and intelligent decision support, directly addressing physician burnout by reducing cognitive load.

Population health management capabilities create opportunities to analyze large-scale health trends and optimize resource allocation. The shift toward value-based care is facilitated by cloud analytics enabling comprehensive outcome measurement and quality improvement initiatives.

Critical challenges require comprehensive approaches combining advanced encryption, robust access controls, and privacy-preserving techniques like federated learning. Interoperability challenges demand standardized data formats and API design protocols. As emphasized by Yang et al. (65), healthcare organizations must implement comprehensive model validation frameworks including bias detection and continuous performance monitoring.

Addressing the digital divide requires targeted efforts supporting smaller organizations through shared services models and technical assistance.

Successful implementation requires holistic approaches addressing technical, organizational, and regulatory challenges simultaneously. Organizations investing in comprehensive change management and gradual implementation strategies achieve better outcomes while minimizing clinical operation disruption.

### 5.1 System implementation

Model development typically follows an end-to-end workflow: First, data is queried from BigQuery, pre-processed in Dataflow, and staged in Cloud Storage buckets from which TensorFlow reads directly. Then hyperparameter tuning and cross-validation are orchestrated with Vertex AI, after which the best-performing checkpoints are exported for online or batch prediction. The scalability advantages of cloud-based training are particularly evident when handling large healthcare datasets. Sierra-Sosa et al. (11) utilized multi-GPU workstations to train deep learning models on 150,000 diabetic patient records, achieving significant performance improvements over traditional machine learning approaches (66). Continuous evaluation dashboards monitor data drift and performance, leading to retraining when clinical data changes or data distributions shift. Case studies indicate that TensorFlow models trained on this stack may improve prediction accuracy for hospital readmission, sepsis onset, or radiographic abnormalities hours earlier than traditional rules-based systems, enabling proactive interventions and resource optimization (12, 13, 67).

The development of a TensorFlow Model for Predictive Analysis requires the use of cutting-edge tools such as Google's Healthcare API, BigQuery, Cloud Machine Learning Engine, and TensorFlow to harness the potential of predictive analytics in the healthcare sector (68). By leveraging these technologies, healthcare institutions can make transformative steps toward enhancing patient care delivery and optimizing resource allocation. The healthcare API plays a pivotal role to accelerate data ingestion, storage, and integration of various healthcare data formats. When combined with tools such as BigQuery and TensorFlow, this API empowers researchers to effectively apply analytics and AI to their datasets and aid them in automatically identifying patterns, accurately predicting clinical outcomes, and efficiently analyzing large datasets (9).

Moreover, the implementation of de-identification techniques is crucial for safeguarding data privacy during the training of TensorFlow models for predictive analysis. Before training, data ingested through the Healthcare API are transformed into analytic-ready tensors. These tensors often include features engineered from structured EHR fields, encoded clinical text, and medical images or scans using pixel-level imaging attributes. De-identification pipelines run in parallel to strip or tokenize protected health information, ensuring datasets comply with HIPAA, GDPR, and local privacy mandates throughout the modelling lifecycle. During training, encryption at rest and in transit, fine-grained IAM roles, and VPC-Service Controls isolate and protect sensitive workloads, allowing data scientists to experiment without exposing patient identifiers (11, 67).

In addition, the Cloud Machine Learning Engine provides the computational backbone for developing and deploying advanced TensorFlow models. When healthcare organizations train models on this platform, they can scale seamlessly from experimental prototypes to production workloads while taking advantage of optimized GPU and TPU resources. This enables rapid iteration on architectures designed to identify patient-specific risk factors, predict disease progression, and recommend personalized treatment regimens that are derived from multimodal clinical data.

As the regulatory landscape evolves and new privacy-enhancing technologies mature, healthcare institutions will increasingly pair these safeguards with TensorFlow pipelines to fully harness the potential of predictive analytics while adhering to strict data privacy regulations (12, 13, 67).

De-identification methods play a critical role in protecting data privacy within the healthcare sector, particularly when using the Cloud Healthcare API for predictive analysis. Through the implementation of de-identification processes, personally identifiable information such as PHI can be masked, removed, or anonymized within datasets. This practice is vital for various purposes, including sharing health information with authorized parties, generating and evaluating datasets from diverse sources, and anonymizing data for use in machine learning models (69, 70).

An important consideration in employing de-identification techniques is the separation of electronic protected health information (ePHI) from de-identified data. The Cloud Healthcare API offers functionalities that extract information from an initial dataset and transfer the results to a newly designated dataset specified by the user. This method allows for the isolation of sensitive data that require protection while still providing access to de-identified data for research, analytics, or predictive modeling objectives (71). Furthermore, de-identification procedures are crucial for adhering to regulations such as HIPAA and GDPR that govern the security and privacy of patient data. Given the significant financial penalties and settlements exceeding $60 million for HIPAA violations recorded between 2018 and 2021, healthcare entities must prioritize measures related to data privacy and security (72). De-identification helps mitigate the risk of data breaches and unauthorized access by ensuring that sensitive information remains protected throughout data processing and analysis (20).

In summary, implementing robust de-identification methods is strictly necessary for upholding data privacy when leveraging the Cloud Healthcare API for predictive analytics in the healthcare sector. By segregating identifiable details from de-identified data, healthcare institutions can maintain patient confidentiality while benefiting from valuable insights gleaned from their datasets.

### 5.2 Successful implementation analysis

Numerous healthcare institutions have successfully integrated the Cloud Healthcare API, capitalizing on its functionalities to improve both patient care and operational efficiency (73). One notable example is the deployment of AI-powered, cloud-based solutions in clinical environments, which has been associated with reductions in physician burnout and improvements in decision support (74).

National health systems have also effectively implemented FHIR-compliant, API-based connectivity across multiple facilities, ensuring secure access to patient data regardless of location. This approach significantly improves the availability and accessibility of essential medical information (75).

Furthermore, large hospitals have advanced their use of cloud-based healthcare APIs by leveraging standardized interoperability protocols to unlock the full potential of underlying EHR systems. This secure and scalable infrastructure empowers clinicians with advanced analytics and machine learning models, producing actionable insights that directly inform and enhance patient care delivery (76).

These examples illustrate the diverse applications of cloud-based healthcare APIs across various healthcare environments. From real-time insight delivery and enhanced system interoperability to predictive analytics capabilities, these implementations highlight the transformative impact of adopting robust, cloud-based solutions to drive better patient outcomes (77).

### 5.3 Healthcare impact and transformation

Cloud-based healthcare solutions utilizing artificial intelligence and advanced analytics have demonstrated substantial capabilities in addressing healthcare challenges. The integration of these technologies creates unprecedented opportunities for improving patient outcomes, operational efficiency, and care delivery models across diverse healthcare settings.

Cloud-based predictive analytics platforms have emerged as powerful tools for reducing hospital readmissions, particularly for patients with chronic diseases. Hospital readmissions represent significant financial burdens for healthcare organizations and indicate suboptimal patient outcomes and care transitions. The implementation of Extract, Transform, and Load (ETL) systems through AI-enhanced cloud enable healthcare organizations to process vast amounts of patient data in real-time, identifying high-risk patients before discharge and implementing targeted interventions. These systems analyze multiple data streams including electronic health records, laboratory results, medication histories, and social determinants of health to create comprehensive risk profiles. As demonstrated in recent systematic reviews, cloud-based healthcare services offer enhanced accessibility to medical records, improved collaboration among healthcare professionals, cost savings on IT infrastructure, and better scalability and flexibility for processing large datasets (78, 79). Healthcare organizations utilizing cloud-based predictive models report significant improvements in readmission reduction, with some implementations achieving up to 37% decreases in 30-day readmission rates for heart failure patients (80). The scalability of cloud infrastructure allows these predictive models to continuously learn and adapt, incorporating new patient data and refining risk stratification algorithms to maintain accuracy across diverse patient populations. This technological foundation supports the development of comprehensive risk assessment frameworks that can identify high-risk patients before discharge and trigger appropriate interventions (81).

Additionally, Cloud-based platforms provide the computational infrastructure necessary for implementing sophisticated machine learning algorithms in readmission prediction. These systems can process petabyte-scale datasets and apply complex algorithms to identify patterns and risk factors that may not be apparent through traditional analysis methods (82). The integration of artificial intelligence and machine learning with cloud infrastructure has shown particular promise in disease detection and treatment applications, which directly contribute to readmission prevention efforts (83). Healthcare providers can utilize these technologies to develop personalized risk profiles for individual patients, enabling targeted interventions based on specific risk factors and clinical characteristics.

Furthermore, Cloud-based solutions facilitate the implementation of remote patient monitoring and telemedicine services that are crucial for preventing readmissions. The elastic scalability of cloud platforms allows healthcare organizations to rapidly expand telehealth services during periods of high demand, such as during the COVID-19 pandemic, while maintaining consistent performance and security standards (80). As highlighted in current literature, cloud computing enables the delivery of telemedicine services and remote patient monitoring solutions, allowing healthcare providers to remotely diagnose, treat, and monitor patients in real-time (79). The integration of real-time monitoring capabilities through IoT devices and wearable technology enables continuous patient surveillance post-discharge, with AI algorithms detecting early warning signs of deterioration and triggering preventive interventions before readmission becomes necessary (84). Healthcare organizations implementing cloud-based remote monitoring report significant improvements in chronic disease management, with patients experiencing reduced hospitalization rates and improved medication adherence. In addition, cloud-based telemedicine solutions support advanced analytics capabilities that can identify population health trends and optimize resource allocation for remote care programs (75).

The deployment of cloud-based solutions has shown remarkable potential in addressing physician burnout by automating routine administrative tasks and streamlining clinical workflows. AI-enhanced cloud platforms significantly reduce the documentation burden on healthcare providers through automated clinical note generation, data entry, and decision support systems. Healthcare organizations implementing these solutions report substantial reductions in time spent on administrative tasks, with physicians experiencing average time savings of 27.4% following implementation of cloud-based data processing systems (80). The automation capabilities extend beyond documentation to include intelligent scheduling, automated quality assurance, and predictive resource allocation, enabling healthcare providers to focus more time on direct patient care. Cloud-based platforms also provide real-time access to comprehensive patient information across multiple care settings, reducing the cognitive load associated with information gathering and synthesis. The implementation of these systems has demonstrated measurable impacts on provider satisfaction and job performance, with healthcare organizations reporting improved physician retention rates and reduced burnout scores following deployment of comprehensive cloud-based clinical support systems (85).

## 6 Future research directions

The advancement of healthcare APIs and interoperability may facilitate increased collaboration collaboration among healthcare API companies, governmental agencies, and international organizations to establish global standards and best practices for healthcare data exchange and system integration. This evolution will increasingly focus on addressing the dimensions of healthcare big data including volume, velocity, variety, and veracity while ensuring that deep learning frameworks can effectively process diverse healthcare data types. The shift toward greater standardization, enhanced model interpretability, widespread adoption of cutting-edge technologies, and a more patient-centered approach to care indicates a promising future for the industry (86).

With the exponential growth in clinical and research data generated by various biomedical tools such as genomics, mobile biometric sensors, and smartphone apps, analyzing this vast amount of heterogeneous data has become crucial for gaining further insights into procedural and technical enhancements in healthcare. Future developments will increasingly leverage advanced deep learning architectures, including convolutional neural networks for medical imaging, recurrent neural networks for sequential health records, and autoencoders for unsupervised feature learning from genomic data. These approaches can automatically learn complex and robust features from raw data without requiring manual feature engineering, addressing the traditional limitations of machine learning methods that rely heavily on domain expertise. The analysis of data sourced from EHRs, medical devices, claims data, and patient-generated information is continuously contributing to the development of robust prognostic frameworks. This collaborative analysis aims to lower analytical costs, create effective clinical decision support (CDS) systems, establish platforms for improved treatment strategies, and identify and prevent fraud associated with big data (63).

The integration of computational systems for signal processing by both researchers and clinical practitioners has experienced significant growth, with particular emphasis on addressing the challenges of data insufficiency, privacy preservation, and model interpretability that currently limit deep learning applications in computational medicine. Future research will focus on developing sophisticated models that merge physiological data with “omics” techniques, enhanced by transfer learning and multimodal learning approaches, to deepen our comprehension of disease conditions and lead to innovative diagnostic tools. As noted by Yang et al. (2021), the field must address critical challenges including insufficient data, model interpretability, data privacy, and heterogeneity to fully realize the potential of deep learning in healthcare applications (65).

Microsoft's strides in cloud technologies for healthcare with Azure Health Data Services underscore the leveraging of trusted AI to tackle transformative challenges while enhancing patient engagement and clinician experiences through an integrated cloud offering. Collaborations between entities such as HCA Healthcare and the Cloud focus on analytics-driven process enhancements that provide clinicians with deeper insights by integrating clinical decision support, leading to improved quality safety efficiency (64, 87, 88). Interpretability plays a pivotal role in predictive analytics in different sectors such as healthcare (79), social media engagement (89) and churn prediction in banking sector (90).

Future trends in predictive analytics will increasingly center around the concept of Compositional AI which enables the seamless composition of existing AI/ML services to create new composite services capable of addressing complex multi-domain healthcare use-cases. This approach promotes reuse, agility, and efficiency in development and maintenance efforts by allowing healthcare organizations to leverage existing models and datasets for new applications. The integration of DataOps and MLOps pipelines will become crucial, enabling deployed ML models to provide training datasets for new models, thereby creating a continuous learning ecosystem. Innovative platforms such as Snowpark's ML modeling API (91), support real-time insights through predictive algorithms while addressing ethical and regulatory considerations. Harnessing tools such as FHIR RESTful APIs combined with compositional AI frameworks will facilitate the development of innovative solutions that allow healthcare professionals to extract meaningful insights from patient data, ensuring better care delivery and enhanced health outcomes while propelling advancements within the industry forward.

## 7 Conclusion

The way healthcare organizations access, manage, and utilize clinical data has evolved significantly, exemplified by the Google Cloud Healthcare API (92). Its interoperability, integration with diverse data sources, and connection to advanced analytics tools including BigQuery, Cloud Dataflow, and machine learning engines create an ecosystem that supports predictive analytics and intelligent care delivery (93). Successful implementations have demonstrated measurable reductions in hospital readmissions, enhanced operational efficiency, and improved patient satisfaction (82). Predictive analytics features supported by scalable cloud infrastructure and advanced machine learning frameworks such as TensorFlow have proven key to modern healthcare transformation (94). The integration of AI with cloud computing has demonstrated effectiveness in addressing critical healthcare challenges, including physician burnout through workflow automation, improved care coordination through enhanced interoperability, and expanded access to care through telemedicine and remote monitoring capabilities.

However, healthcare organizations must carefully evaluate their reliance on cloud service providers and develop strategies to mitigate risks that could limit future flexibility, risk data integrity and increase long-term costs. Healthcare organizations implementing cloud-based imaging solutions must invest in high-performance networking infrastructure and optimized data compression techniques to maintain clinical workflow efficiency (95). The organizations should implement phased deployment strategies, utilize open standards, and develop change management programs. Investment in technical infrastructure and staff training represents a critical success factor. Organizations that address these challenges while leveraging cloud capabilities may achieve improvements in patient outcomes, operational efficiency, and healthcare innovation while maintaining the flexibility and security necessary for sustainable healthcare delivery in an increasingly digital environment (2).

## Footnotes

^1^ https://build.fhir.org/resourcelist.html
